# Social behaviour in bees influences the abundance of *Sodalis* (Enterobacteriaceae) symbionts

**DOI:** 10.1098/rsos.180369

**Published:** 2018-07-11

**Authors:** Benjamin E. R. Rubin, Jon G. Sanders, Kyle M. Turner, Naomi E. Pierce, Sarah D. Kocher

**Affiliations:** 1 Lewis-Sigler Institute for Integrative Genomics, Princeton University, Princeton, NJ, USA; 2 Department of Pediatrics, University of California San Diego, La Jolla, CA, USA; 3 Department of Organismic and Evolutionary Biology, Harvard University, Cambridge, MA, USA; 4 Howard Hughes Medical Institute, Harvard University, Cambridge, MA, USA

**Keywords:** sweat bees, eusociality, microbiome, *Sodalis*, Halictidae, symbiosis

## Abstract

Social interactions can facilitate transmission of microbes between individuals, reducing variation in gut communities within social groups. Thus, the evolution of social behaviours and symbiont community composition have the potential to be tightly linked. We explored this connection by characterizing the diversity of bacteria associated with both eusocial and solitary bee species within the behaviourally variable family Halictidae using 16S amplicon sequencing. Contrary to expectations, we found few differences in bacterial abundance or variation between social forms; most halictid species appear to share similar gut bacterial communities. However, several strains of *Sodalis,* a genus described as a symbiont in a variety of insects but yet to be characterized in bees, differ in abundance between eusocial and solitary bees. Phylogenetic reconstructions based on whole-genome alignments indicate that *Sodalis* has independently colonized halictids at least three times. These strains appear to be mutually exclusive within individual bees, although they are not host-species-specific and no signatures of vertical transmission were observed, suggesting that *Sodalis* strains compete for access to hosts. The symbiosis between halictids and *Sodalis* therefore appears to be in its early stages.

## 1. Introduction

Close contact between individuals of social species provides a means of continual exchange of associated microbes. Indeed, social interactions between hosts substantially impact the composition of bacterial communities associated with those individuals, reducing variation between interactors in honeybees, birds, baboons and humans [1–4]. Such microbial sharing has even prompted theory suggesting that the advantage of transmitting beneficial microbes could itself select for the evolution of social behaviour [5,6]. However, the role that social behaviour plays in shaping microbial communities (and perhaps even the reverse) remains uncertain.

Hymenoptera (bees, ants and wasps) are an ideal model for studying the feedback between sociality and bacterial communities. Social behaviour has evolved a number times in this group, giving rise to the highly eusocial ants and honeybees, but it has also been repeatedly lost in some clades, generating substantial variation in social structure. All hymenopteran species provision their young with food [7,8], potentially allowing for some transmission of bacteria from mother to offspring, but the repeated interactions among individuals in social colonies, including trophallaxis in adults in many species [7], generate more opportunities for microbial transfer within social taxa.

Most work on social insects and their microbiota has examined honeybees and bumblebees, where the microbial communities present appear to be host-specific and consistent across individuals and generations [9–11]. In these bee species, strong evidence points to the importance of social behaviour in establishing and maintaining these bacterial taxa throughout evolutionary history [1,12–14]. Despite this dependence on social interactions to transmit symbionts, the few existing comparisons of bacterial communities between social and solitary bees have found little evidence for an effect of social structure [15,16]. However, these studies focused on two socially polymorphic species, *Megalopta centralis* and *M. genalis*, in which females from a single population can produce either social or solitary nests, limiting the potential for co-evolution between host behaviour and microbial community. A study focused on the evolution of Hymenoptera-associated *Lactobacillus* showed consistent results: host specificity with honeybees and bumblebees but more generalized host use in other bees and ants [17]. Large studies across the ants have also shown huge differences in the degree of dependence and specialization between bacteria and hosts [18], suggesting that bacterial community dependence is quite plastic in Hymenoptera.

The hypothesis that specialized symbioses originate more readily in social compared to solitary taxa [5,6] has also received little attention. Obligate symbionts are common across insects and many are only able to survive inside the specialized organs or cells of their hosts [19,20]. The best studied of these symbionts are the *Buchnera* inhabiting aphid species [19,21], many of which are communal or even eusocial, but similar relationships span a diversity of insects including flies [22,23], weevils [24–26] and the eusocial ants [20,27]. Although a number of the taxa identified as important members of the eusocial honeybee and bumblebee gut communities appear to be host-specific [15,28], none have yet been described as obligate inhabitants of bees. However, bacterial communities associated with other bee taxa have received far less attention and may include lineages with strict specialization on particular bee hosts. Indeed, bacterial 16S rRNA gene sequencing of the solitary bee *Megachile rotundata* [29] and of pollen provisions from the ‘subsocial' bee *Ceratina calcarata* [30] identified *Sodalis,* a taxon for which nearly every member previously characterized has been found to depend on specific insect hosts. While the single free-living form is potentially a widespread associate of plants [31], its associations with these bees is, nevertheless, quite intriguing.

To study the interaction of social behaviour with bacterial communities and explore the possibility of bee-associated obligate symbionts, we examined the gut communities across eusocial and solitary species of halictid bees. Because eusociality has evolved 2–3 times independently within this clade and has been secondarily lost many more times, halictids have a phylogeny rich in closely related species with different social behaviours [32–34]. We also examined differences in the microbial communities of a single, socially polymorphic species, *Lasioglossum albipes*. Using both these cross-species and intraspecific comparisons, we assess two main questions. First, do the bacterial communities of social hosts differ in composition or variation from those of solitary hosts? And second, is the frequency of obligate symbiosis greater in social hosts than solitary ones?

## 2. Material and methods

### 2.1. Sample collection

**Figure 1.**
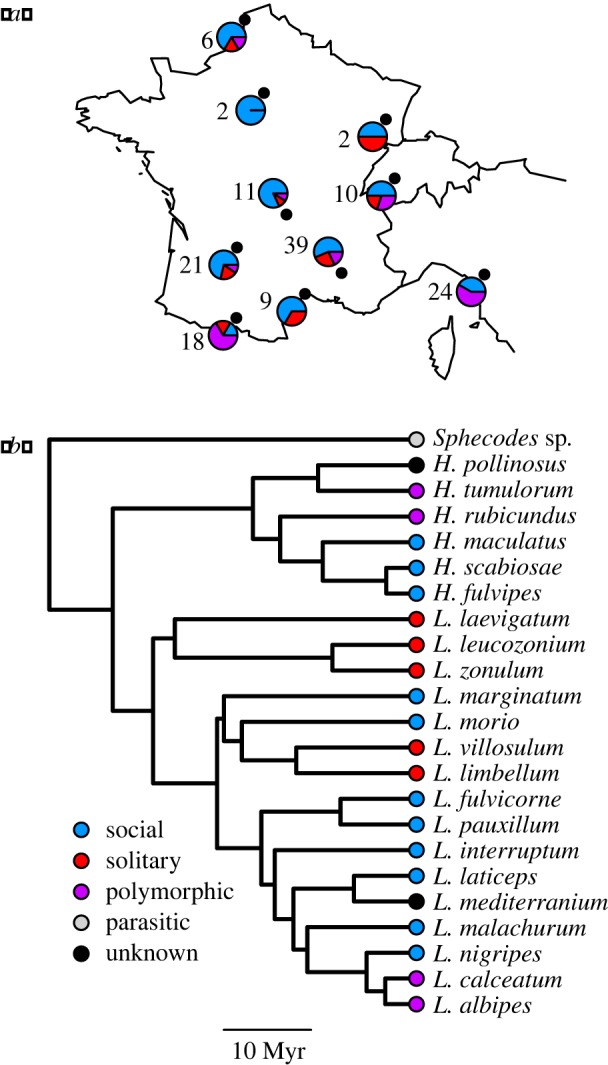
(*a*). Collecting sites for all specimens used for 16S rRNA gene sequencing, number of total specimens from each site, and proportions of those samples classified as each behavioural type. (*b*) Phylogeny of halictid species sampled in this study based on the time-calibrated phylogeny from [33]. Social behaviour is indicated by colours.

We collected 336 bees from Western Europe (figure 1*a*) from three genera (*Lasioglossum, Halictus* and *Sphecodes*) of halictids including 11 eusocial species, five solitary species, four socially polymorphic species (capable of producing both solitary and eusocial nests), and one parasitic species spanning tens of millions of years of evolutionary history (figure 1*b*). Bees were field-caught and immediately flash-frozen over liquid nitrogen. Characteristics of all samples are given in electronic supplementary material, table S1. For most samples, we used whole abdomens for DNA extraction, but guts were also dissected under sterile conditions for 30 specimens including 13 solitary *L. albipes,* 10 *L. malachurum,* two *H. pollinosus*, two *Sphecodes,* and one each of *L. calceatum, L. lativentre* and *L. zonulum*. We used the Mo Bio PowerSoil DNA Isolation Kit (Qiagen, Hilden, Germany) with the addition of proteinase K digestion [35] for all DNA extractions.

### 2.2. 16S Sequence processing

From these samples, we sequenced the bacterial 16S rRNA gene with 150 bp paired-end Illumina reads using the standard approach of the Earth Microbiome Project. These sequences were reference-clustered into 97% operational taxonomic units (OTUs) against the Greengenes database [36] using UPARSE [37] and QIIME [38]. Sequences that failed to cluster against this database were subsequently clustered into *de novo* OTUs. Throughout, OTUs with representative sequences in the Greengenes database are referred to using their index number prefaced by ‘GG’ and *de novo* OTUs are prefaced by ‘denovo'.

### 2.3. Differences in social and solitary bacterial communities

After filtering to include only *Lasioglossum* samples with known social behaviour, excluding all samples from the behaviourally polymorphic species *L. albipes* and *L. calceatum,* and standardizing across samples by rarefying the resulting table to 20 000 reads/sample, we were left with eight social species (*L. fulvicorne* (*N* = 1)*, L. interruptum* (*N* = 2)*, L. laticeps* (*N* = 10)*, L. malachurum* (*N* = 21)*, L. marginatum* (*N* = 1)*, L. nigripes* (*N* = 10)*,* and *L. pauxillum* (*N* = 10)) and five solitary species (*L. laevigatum* (*N* = 2)*, L. leucozonium* (*N* = 16)*, L. limbellum* (*N* = 3)*, L. villosulum* (*N* = 6)*,* and *L. zonulum* (*N* = 1)). We used these data to test for differences in the abundance of taxa between social and solitary samples using Mann–Whitney *U* tests. We tested only the core OTUs present in at least 50% of samples overall as well as those present in at least 50% of samples in each species represented by at least 10 samples. We also tested all taxa making up at least 0.1% of sequences in the overall dataset. *p*-values were corrected for multiple testing using the Benjamini–Hochberg procedure. Social (*N* = 6) and solitary (*N* = 19) forms of *L. albipes* were also compared using Mann-Whitney *U* tests to identify overlapping differences between and within species. To confirm these results, linear mixed models were used to further examine those taxa identified as significantly different in abundance from the Mann–Whitney tests, including geographical region from which they were collected as a random variable.

We hypothesized that social samples would have lower variation in bacterial communities than solitary samples, with additional consistency arising from the shared nests and food resources of the social bees. We used Brown–Forsythe tests to assess differences in the variance of abundance among the core and common taxa in social and solitary bees. We also assessed differences in community dispersion between species and between social and solitary bees using the PERMDISP2 procedure implemented as betadisper in the R vegan package. For both of these analyses, we only included those species for which we had at least 10 samples (four social species and one solitary species) so that we could have confidence in estimates of variance within species. We calculated beta diversity for this analysis using both unweighted and weighted Jaccard.

### 2.4. Classifying by behaviour, location and species

We built automated classifiers using the supervised learning method implemented in QIIME to determine whether bacterial communities were identifiably different between bee species, social behaviours and collecting locality. For classification of social behaviours, we used only *Lasioglossum* species with known records of social or solitary behaviour, excluding the polymorphic *L. albipes* and *L. calceatum*.

### 2.5. Halictid-associated *Sodalis* genome assembly

*Sodalis* was an unexpected yet dominant member of the recovered bacterial communities, so we integrated a number of genomic datasets in order to examine it in greater detail. We extracted *Sodalis* sequences from a shotgun sequencing dataset of 150 adult male *L. albipes* and individuals from 13 other halictid species: *Augochlorella aurata*, *Augochlora pura, Agapostemon virescens, Lasioglossum leucozonium, L. figueresi, L. marginatum, L. vierecki, L. zephyrum, L. calceatum, L. malachurum, L. oenotherae, L. pauxillum* and *Halictus ligatus*. We also downloaded all raw data (excluding mate-pair libraries) sequenced as part of the 10 bee genome project [39] and for the *Ceratina calcarata* genome sequencing project [40] and extracted *Sodalis*-derived reads. These data were used to assemble *Sodalis* genomes *de novo* using metagenomic procedures and infer the evolutionary history of this group (electronic supplementary material, text S1). We used the RAST Web server to annotate functional elements in the *Sodalis* genomes and assessed the possibility of genome-wide relaxed selection by estimating dN/dS ratios in the recovered coding sequences using the free-ratios model of PAML v.4.9 [41,42].

## 3. Results

### 3.1. Relative abundance of bacteria differs little

We aimed to identify differences in bacterial communities of social and solitary bees. So as not to be confounded by differences between genera and polymorphic taxa, this analysis was limited to only those species in the genus *Lasioglossum* that have been verified as either strictly social or strictly solitary. Mann–Whitney *U* tests of the 38 taxa making up at least 0.1% of all sequences in the dataset or present in at least 50% of all samples show that five OTUs are significantly (FDR-corrected *p* < 0.01; electronic supplementary material, table S2) more abundant in solitary bees including one from *Wolbachia* (GG836919) and four from *Sodalis* (GG261110, GG2093965, GG4335746, denovo1)*.* Four OTUs are significantly more abundant in social bees including three from *Wolbachia* (GG273974, GG835499, GG101940) and one from *Sodalis* (GG4316320). However, as geography may impact the bacteria present, we expanded this analysis to include collection locality as a random effect in a linear mixed model. When geography was taken into account, most OTUs were no longer significantly different in relative abundance between social and solitary bees (*p* > 0.05 using Kenward–Roger approximations for degrees of freedom), with the exception of *Sodalis* OTU denovo1 (*t* = 2.4, *p* = 0.046) and *Wolbachia* OTU GG835499 (*t* = 2.5, *p* = 0.048), which remained enriched in solitary and social bees, respectively.

We also tested for differences in the frequency of *Sodalis* within social forms of *L. albipes* through the detection of *Sodalis* reads in an *L. albipes* shotgun genomic resequencing dataset. Of 75 specimens of each behavioural type, only six eusocial samples have detectable levels of *Sodalis* as opposed to 30 solitary samples (electronic supplementary material, text S1). This difference in frequency is significant according to a *χ*^2^ test (*p* = 1.1 × 10^−5^, *χ* = 19.3). Sociality covaries with geographical region in this species, so these results may be confounded by geographical variation. However, *Sodalis* is present in other halictid species from all three of the geographical regions from which these eusocial samples were collected (Rimont, Dordogne and Calais; electronic supplementary material, figure S1), indicating that geography is unlikely to be the factor limiting *Sodalis* colonization.

### 3.2. Sociality does not affect community variability

We tested for differences in variability of individual bacterial taxa using Brown–Forsythe tests of the 129 core and most common OTUs and found that two *Wolbachia* taxa (GG835499 and GG101940) had significantly different variance in social and solitary bees (FDR-corrected *p* < 0.01; electronic supplementary material, table S2). Both of these taxa had higher variance in eusocial samples.

We also compared variance of community assemblage by examining dispersion of weighted (*F* = 3.2, *p* = 0.076) and unweighted Jaccard distances (*F* = 0.38, *p* = 0.54), but did not find significant differences (electronic supplementary material, figure S2B). We also compared the solitary *L. leucozonium* to all eusocial taxa with at least 10 samples using both metrics, but only the comparison with the eusocial species *L. laticeps* showed any indication of a difference in community dispersion using weighted Jaccard (*F* = 4.41, *p* = 0.047).

### 3.3. *Sodalis* dominates community differences among social forms

**Figure 2.**
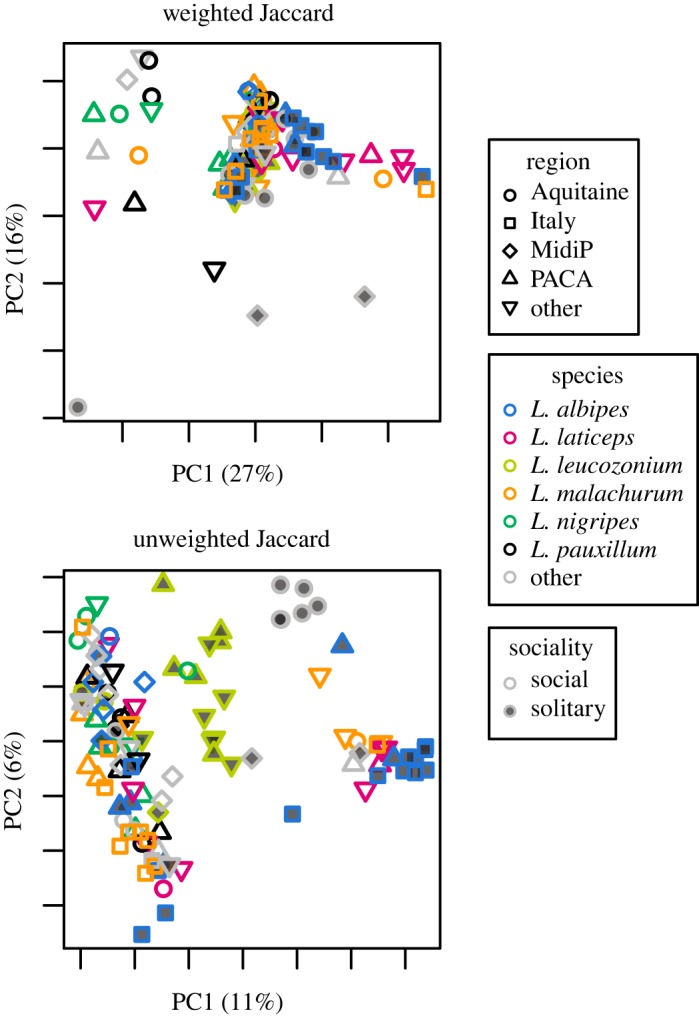
Principal coordinates analysis plots of all halictid samples examined in the current study. MidiP, Midi-Pyrénées; PACA, Provence-Alpes-Côte d'Azur. The most frequently represented geographical regions and species are indicated and others are grouped together. Individual plots represent the use of different beta diversity metrics (weighted and unweighted Jaccard).

No clear differences in overall bacterial communities of bees with different behaviours, from different species or collected at different locations are apparent from principal coordinates analyses (figure 2). We used automated supervised learning classification to determine whether these communities were distinguishable in any way. For the classifier of samples based on social behaviour, the ratio of baseline to observed error was 2.80, meaning that the classifier was 2.8 times better than random guessing. Of the 10 most important features for distinguishing social behaviours, nine are classified as *Sodalis* or the family to which it belongs, Enterobacteriaceae, including three of the most abundant OTUs (GG261110, GG4335746, denovo1). The last OTU in the top 10 is from *Wolbachia* (GG836919). To determine whether other taxa had similar discriminatory power, we removed all 451 OTUs classified as Enterobacteriaceae and reran the supervised learning, which reduced the error ratio to 1.28, indicating that essentially no discriminatory power remained.

From this analysis, we can conclude that *Sodalis* is by far the most important clade distinguishing eusocial and solitary halictids. Indeed, building a classifier based only on the 42 *Sodalis* OTUs is more accurate than one based on all data, with an error ratio of 3.58. Frequency differences of *Sodalis* are apparent between these two groups (electronic supplementary material, figure S3).

### 3.4. *Sodalis* genomes and phylogeny

We recovered large amounts of *Sodalis* data from 36 of the 150 *L. albipes* shotgun sequencing samples as well as six other halictids (*H. ligatus, L. calceatum, L. leucozonium, L. malachurum, L. marginatum* and *L. vierecki*) and *C. calcarata*. These data were used to assemble genomes of the symbionts and infer their phylogenetic history (electronic supplementary material, text S1). Genomes from two strains were recovered from *L. albipes* samples and are abbreviated SAL1 and SAL2. We found SAL1 in *H. ligatus, L. calceatum, L. malachurum, L. marginatum* and *L. vierecki*, in addition to *L. albipes* (electronic supplementary material, figure S4). The *Sodalis* genome recovered from *L. leucozonium* (SLEU) collected in Europe was only found in this species. *Ceratina calcarata* also hosted its own lineage of *Sodalis*.

**Figure 3.**
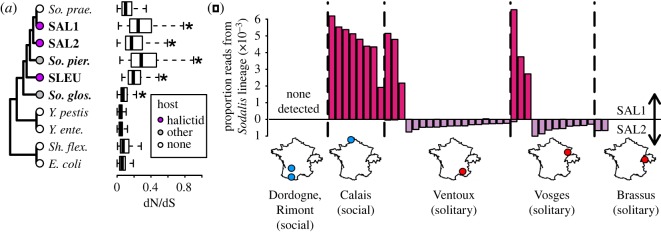
(*a*) Topology showing evolutionary relationships of the three *Sodalis* lineages identified in this study (SAL1, SAL2, SLEU), three previously sequenced *Sodalis* genomes (*So. praecaptivus, So. glossinidius, So. pierantonius*), and several free-living (host: ‘none’) taxa as out-groups. Bolded taxa are symbionts of insects. Boxplots show genome-wide distributions of dN/dS values. dN/dS ratios of *Sodalis* symbionts were compared to *So. praecaptivus* using Wilcoxon rank-sum tests and significant differences are shown with asterisks. (*b*) Proportions of reads originating from the two *Sodalis* lineages SAL1 and SAL2 in shotgun sequencing data for 36 *L. albipes* specimens. Samples are separated by populations of origin shown by maps.

Phylogenetic reconstruction revealed a total of four lineages of bee-associated *Sodalis* (electronic supplementary material, figure S4), and we were able to assemble genomes for three of these (figure 3*a*). The two clades of *Sodalis* present in the *L. albipes* samples do not show any apparent segregation by collecting locality (figure 3*b*). Two of four localities are shared between the two lineages, suggesting that both lineages are widespread and probably interact within the same bee populations.

### 3.5. Co-infections are rare

The finding that two *Sodalis* lineages coexisted in *L. albipes* even within the same geographical populations suggested that these lineages may interact directly. We identified 6407 diagnostic single nucleotide polymorphisms between SAL1 and SAL2, which allowed for confidence in distinguishing these two groups. Although we detect reads from both strains in all but two bees, one strain always dominates, and there are only two individuals in which each strain makes up more than 1% of all *Sodalis* reads. The frequencies of these co-infections with appreciable numbers of reads from both strains are less common than expected by chance (Fisher's exact test *p* = 0.044; figure 3*b*). This pattern could be explained by competition between the two lineages or by vertical transmission of *Sodalis* from mother to offspring, though we find no correlation between mitochondrial lineage of an individual and the strain of *Sodalis* present (electronic supplementary material, figure S4; text S1).

### 3.6. Widespread relaxed selection in *Sodalis*

All *Sodalis* symbiont genomes presented in this study as well as the weevil endosymbiont *S. pierantonius* have significantly higher genome-wide distributions of dN/dS ratios than the only known free-living form of *Sodalis, S. praecaptivus* (*p* < 2.2 × 10^−16^; figure 3*a*), indicating widespread relaxed selection. Notably, the tsetse symbiont *S. glossinidius* instead has significantly lower dN/dS (*p* = 4.2 × 10^−11^). This may be due to the fact that *S. glossinidius* has occupied tsetse flies for a much longer period of time (electronic supplementary material, figure S4), providing opportunity for those genes under relaxed selection to be completely lost.

We also find evidence of gene loss across functional categories in the halictid-associated *Sodalis.* The numbers of genes in each functional subsystem were almost always lower in each of the halictid-inhabiting *Sodalis* taxa than in the free-living *S. praecaptivus*, particularly for SAL1 and SLEU, the two lineages with the highest average dN/dS ratios, smallest total genome sizes and lowest GC content (electronic supplementary material, figure S5; text S1).

## 4. Discussion

Despite research showing the importance of social interactions in the establishment of bacterial communities in honeybees [1], our results suggest that social behaviour has a limited influence on the bacterial communities of social and solitary bees, consistent with previous halictid studies on bacteria [16] (although other halictid associates, such as nematodes, have shown specificity and correlation with sociality [43]). In fact, our cross-species comparisons revealed a consistent association of social structure with the prevalence of only one bacterial taxon: *Sodalis.* Even within a socially polymorphic species, *L. albipes, Sodalis* showed a signature of variation across social forms.

The diversity of insects that play host to *Sodalis* is quite wide, spanning lice, beetles, flies, many hemipterans and bees [22,25,29,44,45]. Where best studied, in the rice weevil *Sitophilus oryzae* and the tsetse fly *Glossina morsitans*, the functional consequences of *Sodalis* symbiosis differ. The weevil symbiont, *Sodalis pierantonius,* plays an essential role in exoskeleton deposition in its hosts [25]. However, no clear function is apparent for *S. glossinidius* in tsetse flies, where the symbionts show comparatively less regular localization (i.e. found in haemolymph) than that seen for weevils (confined to bacteriocytes) [46,47]. Our ability to amplify *Sodalis* from halictid legs and antennae (electronic supplementary material, text S1) suggests that it may also exist as a widespread haemolymph symbiont in bees.

Given the number of different insects occupied by *Sodalis* and the diversity of its consequences in these taxa, it is premature to draw conclusions about its function in bees or the cause of differences in abundance between eusocial and solitary species. Although there are several lines of evidence supporting this finding, additional taxon sampling would be useful as it is possible that the differences in abundance identified are due to species-specific rather than behaviour-specific factors. Nevertheless, it is possible that ‘social immunity’, the group-level behaviours that social species can use to combat pathogens [48], may form the basis of these differences. The significantly more frequent occurrence of *Sodalis* in solitary samples both across halictids and within the socially polymorphic species, *L. albipes*, is consistent with *Sodalis* being more readily eliminated or prevented from establishing in social bees. This apparent antagonism would also imply that the relationship between *Sodalis* and halictids has yet to reach long-term stability.

Including the halictids in this study, at least 12 evolutionarily distinct groups of insects have been separately colonized by *Sodalis*. Several of these lineages show evidence for relaxed selection similar to the patterns we find here, implying nascent obligate symbiosis [24,26,49,50]. Even in comparison to *Wolbachia*, which is estimated to occupy around 20% of insects [51], the flexibility of *Sodalis* is impressive. We find that at least three strains of *Sodalis* have colonized a single genus of halictid, and a fourth strain, apparently spawned from the same ancestral lineage as *S. glossinidius,* occupies *C. calcarata,* a species more closely related to honeybees than to halictids. Yet we find little evidence for host specificity or, given the presence of the same strain in both European and American bees, geographical limitation. Even within a single species of halictid (*L. albipes*) we clearly identified two distinct *Sodalis* lineages, showing a remarkable predisposition of free-living *Sodalis* to infect halictids. Most excitingly, these two *Sodalis* lineages appear to exist in a mutually exclusive way, potentially competing for access to hosts. Here we have added another fold to our understanding of symbiont biology: competition between distinct evolutionary lineages of a nascent symbiont for full access to hosts.

Werren & Windsor [51] proposed that *Wolbachia*'s prevalence indicates a global equilibrium or, alternatively, an ongoing increase in the frequency of *Wolbachia* infection. We propose that a third model may be more appropriate for *Sodalis*. In addition to the four strains identified in bees, the free-living form of *Sodalis* appears to have independently colonized each of the other insect groups with which it associates [24,31,52]. The genomes of each of the lineages that have been sequenced all show widespread relaxed selection, implying that, in every case, *Sodalis* is in the process of becoming an obligate symbiont. However, the frequency with which *Sodalis* infections are established suggests that it may never reach stability as an obligate symbiont, but each lineage may, instead, be eliminated from a given host, either by host immune function, random events or competition with a more recently colonizing strain. Rather than progressing towards stable symbiosis, this may be a continually renewing process wherein new strains are constantly established and eliminated.

Given the relative consistency of halictid-associated bacterial communities regardless of host behaviours, sociality clearly does not lead to a particular set of symbionts. However, we do find evidence that social behaviour influences the abundance of several strains of *Sodalis* both within a single socially polymorphic species and between strictly social and solitary taxa. The mechanism of this impact is, apparently, specific to the conditions of the interactions between *Sodalis* and their hosts as this pattern is unique among the bacteria examined. However, contrary to our prediction that symbiosis would occur more readily among social hosts, the prevalence of *Sodalis* is higher in solitary bees. Unfortunately, until a clear determination can be made as to the nature of the interaction between *Sodalis* and halictids and why abundances differ between bees with different behaviours, we cannot draw any conclusions about whether social evolution is correlated with the presence of beneficial microbes [5,6]. The identification of an apparent incipient symbiont in this socially variable clade of bees does, however, provide a compelling system for understanding the possible role of host social behaviour in this process.

## Supplementary Material

Supplementary text S1

## Supplementary Material

Figure S1

## Supplementary Material

Figure S2

## Supplementary Material

Figure S3

## Supplementary Material

Figure S4

## Supplementary Material

Figure S5

## Supplementary Material

Figure S6

## Supplementary Material

Figure S7

## Supplementary Material

Figure S8

## Supplementary Material

Figure S9

## Supplementary Material

Table S1

## Supplementary Material

Table S2

## Supplementary Material

Table S3

## Supplementary Material

Table S4

## Supplementary Material

Table S5

## Data accessibility

All sequencing data have been deposited in the Short Read Archive of NCBI under BioProject accession number PRJNA402054. OTU representative sequences and *Sodalis* genome assemblies and annotations are available via the Dryad Digital Repository: http://dx.doi.org/10.5061/dryad.0vt7nt0 [53].
