# Supplementary material for: Social behaviour in bees influences the abundance of Sodalis (Enterobacteriaceae) symbionts
Source: R Soc Open Sci. 2018 Jul 11;5(7):180369. doi: 10.1098/rsos.180369 (PMC6083661; doi:10.1098/rsos.180369)
Supplement: Supplementary text S1 [file rsos180369supp1.docx]

**ELECTRONIC SUPPLEMENTARY MATERIAL, TEXT S1**

**Supplementary Methods**

***Quantifying absolute abundance of bacteria***

Triplicate quantitative-PCRs were performed as in Rubin *et al.* [1] and Sanders *et al.* [2]. We took the average of the triplicate measurements for each specimen and standardized these measurements to the concentration of overall DNA present in each sample, measured using a Qubit Fluorometer (Thermo Fischer Scientific, Waltham, MA, USA). Numbers of copies of the bacteria 16S gene per nanogram of DNA was compared between groups using Wilcoxon rank-sum tests.

***16S sequence processing***

Bacterial 16S rRNA gene amplicon sequences were demultiplexed with QIIME [3] and then processed with the UPARSE [4] pipeline to remove low quality and chimeric sequences. The standard operating procedure was followed. Briefly, paired reads were merged, discarding contigs with lengths over 300 bp (median, lower quartile, and the upper quartile were all of length 253) or more than 0.5 expected errors. Singleton sequences were also discarded. Chimera detection was done both with the *de novo* chimera detector built into UPARSE and the UCHIME pipeline using the Greengenes gold database. All filtered sequences were then clustered against these representatives at 97% similarity. Those that did not cluster were discarded.

To compare with other bee microbiomes, we combined our data with two previously reported datasets. Firstly, we drew sequence data from an unpublished Earth Microbiome Project dataset on the honey bee microbiome from Qiita (Study ID: 1064 and in the European Nucleotide Archive under study ID: PRJEB14927). This dataset consisted of 6,744,896 150 base single-end rather than paired-end reads but was otherwise quality filtered using the same procedure as our halictid dataset. We also included the 4,999 bacterial 16S sequences from Martinson *et al*. [5] from GenBank, truncating them to 1,000 bp before clustering.

This combined dataset was open reference clustered against the August 2013 release of the Greengenes database using usearch61 as implemented in QIIME with chimera detection deactivated. Taxonomy for *de novo* OTUs was assigned using RDP [6], sequences were aligned against the Greengenes database using PyNAST [7] and inserted into the reference Greengenes [8] tree using ParsInsert (https://sourceforge.net/projects/parsinsert/). OTUs classified as chloroplast or mitochondria (368) or that failed to align to the Greengenes database (661) were discarded, as were those not represented by at least two sequences (1,663). Halictid samples not represented by at least 20,000 of the remaining reads were also discarded. The OTU table used for all analyses is included as Table S3. The final OTU table includes 7,053 OTUs. After filtering to just halictid samples, 2,039 OTUs remain.

***Behavioral classification***

Behavioral information for the bee taxa examined here was drawn from [9] and references therein. However, we treated *L. fulvicorne* as social rather than solitary [10]. Behaviors of the species *L. mediterrraneum* and *L. pollinosus* have not been previously well-characterized, so they were not included in comparisons of social behaviors.

***Supervised learning classification***

We used automated supervised learning to identify differences between groups of samples. In this approach, OTUs of samples are used to construct models that predict sample groupings based on those OTUs [11,12]. Error was estimated using 10-fold cross validation, splitting the data into 10 sets with approximately equal group membership and using 9/10 sets to build the classifier and predict membership in the last set. Model accuracy is assessed using the estimated generalization error: the ratio of the proportion of classification errors that would occur if guesses were completely random to the proportion of errors from the model. Higher error ratios mean that the classifier is performing better. An error ratio of ≥ 2 is typically used to indicate that there are significant differences in communities [11,12]. An error ratio of 1 indicates that a classifier is no better than random and that OTUs do not differ predictably between groups. All analyses were conducted on tables with OTU quantities represented as proportions so that data were not lost during rarefaction.

In addition to the automated classification of samples based on social behavior, we also built a classifier to distinguish species. The same data as for the social classifier was used for this analysis with the additional constraint that all species included had to be represented by at least 10 samples. This limited the dataset to representatives of *L. laticeps, L. leucozonium, L. malachurum, L. nigripes,* and *L. pauxillum*. We also built a classifier for collection locality, again limiting the dataset to those regions represented by at least 10 samples (i.e. Aquitaine, Florence, Midi-Pyrénées, and Provence-Alpes-Côte d'Azur). Lastly, we used all *Lasioglossum* and *Halictus* data to classify genera.

***Alpha diversity***

We calculated alpha diversity as observed counts of OTUs and the Chao1 index calculated on 100 replicate OTU tables rarefied to 20,000 reads and tested for differences between social and solitary *Lasioglossum* samples using Wilcoxon rank-sum tests, again excluding the polymorphic and uncharacterized species. We calculated rarefaction curves using mothur version 1.36 [13].

***Identification of bacterial sequence in the* L. albipes *genome assembly***

We used the previously published *Lasioglossum albipes* genome to help filter bacteria-derived sequencing reads from several shotgun bee sequencing datasets. However, we first needed to filter scaffolds of bacterial origin from the assembly. Therefore, we downloaded all 4,098 bacterial genomes in the NCBI database labeled as complete (ftp://ftp.ncbi.nlm.nih.gov/genomes/refseq/bacteria/assembly_summary.txt) as of January 7th, 2016. Version 2 of the *L. albipes* genome [14] was downloaded from hymenopteragenome.org. BLASTN was used to query all *L. albipes* scaffolds against a database composed of all of the bacterial genomes, limiting hits to e-values ≤ 1x10^-5^. The resulting hits were then examined for matches against a database of seven animal genomes (*Anopheles gambiae, Danio rerio, Daphnia pulex, Drosophila melanogaster, Gallus gallus, Monodelphis domestica,* and *Mus musculus*). Any sequence with a greater percent similarity to the animal genomes was assumed to be a conserved, slow-evolving sequence. Any scaffold for which more than 25% of its sequence was confidently determined to be bacterial in origin was assumed to be bacterial in its entirety.

**Sodalis *phylogeny***

In addition to the 150 shotgun sequencing *L. albipes* samples (Table S4), we made use of previously collected sequencing data for 12 other halictid species: *Augochlorella aurata*, *Augochlora pura, Lasioglossum leucozonium, L. figueresi, L. marginatum, L. vierecki, L. zephyrum, L. calceatum, L. malachurum, L. pauxillum,* and *Halictus ligatus*. Most samples were collected in France but *L. figueresi* was collected in Costa Rica and *Augochlorella aurata, Augochlora pura, L. vierecki,* and *L. zephyrum* were collected in the northeastern USA*.* For each of these species, we had HiSeqX paired-end 150 bp data collected from 10x Genomics libraries from single individuals. Genomes for each of these bee species were assembled using the Supernova v1.0 pipeline available from 10x Genomics. Scaffolds of bacterial origin were removed from these assemblies as described above for *L. albipes.* We then trimmed the first 24 bases off of the first read (which includes the 10x barcode and spacer sequence) from each of these datasets, collecting those reads that failed to map to the bee genome assemblies and included these unmapped reads in the phylogenetic analysis of halictid-inhabiting *Sodalis*.

We then downloaded seven Enterobacteriaceae genomes and all coding sequences from GenBank: *Yersinia pestis* (NC_003143.1), *Y. enterocolitica* (NC_008800.1), *Escherichia coli* (NC_000913.3), *Shigella flexneri* (NC_004337.2), *Sodalis glossinidius* (NC_007712.1), *So. pierantonius* (NZ_CP006568.1), and *So. praecaptivus* (NZ_CP006569.1). We aligned these genomes with Mauve and mapped our sets of reads that failed to map to bee genomes to the genome-aligned *So. pierantonius* using BWA [15] and called genotypes using FreeBayes [16], specifying ploidy of one, requiring coverage of at least two to call a site, and reporting monomorphic sites. We used a custom python script to create consensus sequences from the resulting genotype data, ignoring indels and any other variants not classified as SNPs by FreeBayes. To be included in further analyses, at least 95% of the length of the genome alignment had to be called for the consensus sequence. The resulting aligned sequences were then used to create a maximum likelihood phylogeny using RAxML v7.3.0 [17] specifying a GTRGAMMA model of nucleotide substitution.

**Sodalis *genome assembly***

The recovered phylogeny showed the existence of two distinct clades of *Sodalis* within *L. albipes* (SAL1 and SAL2)*.* In order to obtain the best possible genome sequences from each of these clades, we assembled the unmapped reads from the individual samples with the highest number of reads mapped to *S. pierantonius*. Metagenomic assemblies of unmapped reads were created using SOAPdenovo2 [18], Ray [19], and the uneven depth version of IDBA [20]. IDBA automatically integrates over several kmers to produce a final assembly and the maximum kmer size was specified as 90. For the other two assemblers, separate assemblies were created using kmers of 31, 41, 51, and 61. All assemblies were then combined using MeGAMerge [21] with a coverage cutoff of 2. *Sodalis* genomes were then extracted from these population level assemblies using the suggested approach of multi-metagenome [22]. To obtain differential coverage depth information, we mapped the data from the samples of origin for each assembly to those assemblies, contrasting them with the sample with the second highest number of *Sodalis*-mapped reads from each clade. We used a GC cutoff of at least 47% to consider a scaffold a part of the target *Sodalis* genome. We also colored our plots using classes rather than phyla to further distinguish *Sodalis* and *Wolbachia* which are both Proteobacteria*.* Reads were extracted from the scaffolds of interest and reassembled using SPAdes [23], optimizing the level of coverage for each assembly by running assemblies for coverages ranging from 10 to 400 for SAL1 and from 10 to 110 for SAL2. We then ran GapFiller [24] on the final genomes and discarded scaffolds of less than 500 bases. Finally, we extracted ORFs at least 90 nucleotides long between start and stop codons using EMBOSS getorf (http://emboss.sourceforge.net/apps/cvs/emboss/apps/getorf.html) and used DIAMOND [25] to find the best hit against the NCBI database of nonredundant bacterial proteins. All scaffolds with fewer than three ORFs with best hits to Gammaproteobacteria were discarded from the assemblies.

The phylogeny also revealed that the lineages inhabiting *L. leucozonium* may be unique and that additional information may be available in the 10x data from the other species. We, therefore, endeavored to assemble *Sodalis* from *L. leucozonium* and *L. calceatum* *de novo*. After some exploration, we determined that different assembly methods would be optimal for each dataset.

For the lineage inhabiting *L. calceatum*, we mapped 10x reads with the first 24 bases trimmed from the first read to the *L. calceatum* genome. Unmapped reads were assembled using both IDBA_UD and Supernova v1.1.0. The Supernova output was constructed in the “pseudohap” style. These two assemblies were concatenated and then all ORF’s with minimum size of 90 nucleotides were extracted using getorf in “find” mode 1 (to identify protein sequences between start and stop codons). We then used DIAMOND [25] to query these ORF’s against NCBI’s nonredundant bacterial protein database, taking just the best hit for each ORF. Hits were only considered if their alignments were greater than 100 residues long and more than 60% identical. Scaffolds for which less than 30% of the ORF’s best hits were to proteins from Gammaproteobacteria were discarded. Finally, scaffolds with less than 40% GC-content were also discarded. The trimmed 10x data was then mapped to the resulting set of scaffolds. Mapped reads were then randomly downsampled to one million reads in five iterations and, for each set of random reads, an assembly was executed using Supernova v1.1.0. The same set of previously used filters for identifying high confidence *Sodalis-*derived sequence via ORF matching was done for each of these assemblies and gaps were closed using GapFiller with the full set of original trimmed 10x reads. These five assemblies were then consecutively merged using Quickmerge [26] with parameters hco=5, quickc=2, minlen=10000, and seedlen=20000. This assembly was then merged with the *L. albipes*-derived assembly for SAL1 as the “hybrid” assembly and with hco=3, c=1, lm=1000, and l=10000. Finally, we mapped all of the reads that went into the Supernova assemblies to this merged assembly using Longranger v2.1.2 and performed a final round of scaffolding using fragScaff [27]. After some parameter exploration, we used -b 1 -E 3000 -G R -j 3 for the final assembly.

Our assembly procedure for the lineage inhabiting the specimen of *L. leucozonium* for which we obtained 10x Genomics sequencing was slightly different. First, we used all reads that mapped to Gammaproteobacteria scaffolds when creating the second round of assemblies rather than limiting the sampling to one million reads. Second, only one iteration of the final Supernova assembly was conducted. Data was far more limited for this genome so our attempts at merging multiple assemblies were unsuccessful. Genome completeness for all assemblies was determined using CheckM [28].

***Identifying coinfections***

The aligned set of consensus genomes was examined to identify SNP’s fixed between the two *Sodalis* lineages assembled from *L. albipes*. We required that genotypes were available for all *L. albipes* individuals with detectable levels of *Sodalis* and that no gaps or unknown bases were present 100 bases up- or downstream for a SNP to be used. In order to get an accurate estimate of the frequency of coinfections by both lineages, we examined individual read pairs for the presence of these SNPs. Read pairs were only counted as representing a particular lineage if at least two SNPs were present and consistent with a single lineage and there were no conflicting genotypes. We only examined read pairs mapped to a single site with no supplementary alignments and that did not require insertions or deletions to be mapped. We also excluded SNP sites that had coverage more than twice the standard deviation greater than or less than the mean coverage at these SNPs.

***Gene tree inference***

We endeavored to confirm that our phylogeny was indeed the most likely by examining gene trees. We downloaded all coding annotations for each of the species used in the phylogenetic analysis from GenBank as well as two additional taxa: *Vibrio cholerae* (NC_002506) and *V. parahaemolyticus* (NC_004603). We created annotations of the *Sodalis* genomes assembled here by uploading them to the RAST server [29]. Orthologous groups were determined using Proteinortho [30] with a minimum connectivity of 0.8. These orthologous groups were aligned with Prank [31] and gene trees were inferred for all loci represented by at least four taxa using RAxML v7.3.0 and the GTRGAMMA model of nucleotide substitution. This set of gene trees was examined using ASTRAL [32–34].

Once the most likely species tree was determined, we repeated the phylogeny inference procedure used above. However, this time the Mauve alignment included the four *Sodalis* genomes assembled here and the inferred phylogeny was constrained to the topology inferred from the gene tree inference.

***Relaxed selection***

To quantify the degree of relaxed selection in the *Sodalis* genomes assembled here, we used the free ratios model in PAML v4.9 [35,36] to estimate dN/dS ratios for all orthologous groups represented by at least four taxa. We required that aligned loci be at least 300 bases long, that dN/dS ratios be estimated as less than 20 and greater than 0.0001 and that the estimated dS value be at least 0.001. These quality controls were particularly important given that several of the terminal branch lengths are quite short. Statistical differences between distributions of dN/dS ratios were calculated using Wilcoxon rank-sum tests. We also had interest in determining the degree of relaxed selection in previously discovered and potentially obligate endosymbionts of honey bees. We, therefore, examined three honey bee endosymbionts with fully sequenced genomes and compared each of them to three of their close relatives using the same methods.

**Sodalis *genome function***

Genes were assigned subsystem functions using the RAST server. The counts of genes assigned to each subsystem for the free-living *S. praecaptivus* genome were used as the baseline expected number of genes. Each newly identified *Sodalis* lineage was compared to this baseline.

***Inheritance pattern***

We examined possible patterns of maternal inheritance of *Sodalis* in the *L. albipes* specimens by identifying the mitochondrial lineage of each *L. albipes* sample. We identified the *L. albipes* genome scaffold corresponding to the mitochondrial genome by downloading all of the mitochondrial protein sequences present in the honey bee mitochondrion (GenBank accession KX870183 on May 17, 2017) and querying these against the *L. albipes* assembly. We calculated genotypes for this scaffold using the best practices workflow of GATK [37–39]. We then inferred haplotype information across the mitochondrion for the 36 *L. albipes* found to host *Sodalis*.

***PCR localization***

Identifying the location within the body of *Sodalis* infection can help to elucidate its functional consequences. Therefore, we developed *Sodalis*-specific diagnostic primers based on the 16S sequence of *S. praecaptivus* (JX444565.1) with emphasis on the exclusion of the most common *Wolbachia* taxon (Greengenes taxon 273974). This *Wolbachia* lineage is very similar to that which occupies *Drosophila simulans* and has been fully sequenced (GenBank CP001391.1). We developed the primers BRsod1104F (5’-TTCGGTCGGGAACTCAAAGG-3’) and BRsod1290R (5’-AGTTGCAGACTCCAATCCGG-3’). The numbers indicate coordinates based on the *S. praecaptivus* 16S gene. PCR’s were done in 10µl reactions with 5µl of OneTaq 2X master mix (NEB, Ipswich, MA, USA), 2µl lab grade water, 1µl of each primer at 10µM concentration, and 1µl DNA extract. Temperature cycling was done as follows: denature for three minutes at 95 ºC followed by 35 cycles of 30 seconds at 95 ºC, 30 seconds at 60 ºC, and 45 seconds at 72 ºC and a final extension at 72 ºC for five minutes. We confirmed that the product of these primers was *Sodalis-*derived by Sanger sequencing (GENEWIZ, South Plainfield, NJ, USA).

We attempted to localize *Sodalis* in halictid bodies by extracting DNA from five body parts (head, thorax, abdomen, antennae, legs) of three male *L. albipes* and attempting to amplify this *Sodalis* locus from each of these parts. DNA extractions were done using the Qiagen Blood & Tissue DNeasy kit (Qiagen, Hilden, Germany) and results of the PCR were visualized using gel electrophoresis. We ran PCR’s of the COI barcoding locus using the primers Jerry and Pat (Magnacca & Danforth 2006) on all of the same DNA extractions to confirm DNA quality. All three negative control PCR’s that included all reagents but no DNA extract yielded no PCR product.

**Supplementary Results**

***Bacterial 16S sequencing***

Of 9,943,931 initial read pairs from the halictid 16S amplicon sequencing, 8,259,061 remained after quality controls, including 126,833 unique sequences. The automated chimera detection of UPARSE deemed 12,403 sequences to be chimeras. The remaining sequences clustered into 1,109 OTU’s of which 13 were determined to be chimeras using UCHIME, leaving 1,096 non-chimeric OTUs. All filtered sequences were then clustered against these representatives at 97% similarity leading to 98% of quality filtered reads clustering with these high quality, non-chimeric sequences for all subsequent analyses. All raw sequence reads have been deposited in NCBI’s Short Read Archive under BioProject accession PRJNA402054.

***Few taxa dominate the halictid microbiome***

We generated a total of 9.9 million read pairs from halictid microbial communities. Rarefaction curves indicate that our specimens were well-sampled (Fig. S5). The vast majority (97.9%) of sequences recovered from halictids were represented by only the 25 OTUs making up more than 0.1% of total sequences (7,543,271/7,701,949) (Fig. S6). Among these, three are clear outliers. OTU GG273974 (*Wolbachia*)*,* makes up 51.8% of all sequences and taxon GG829017 (*Lactobacillus*) makes up 17.9% of sequences. Lastly, taxon GG4316320 (*Sodalis*) makes up 9.3% of all sequences. All other taxa each make up less than 4% of total sequences.

Of the 25 abundant OTUs, 16 were also core OTUs (present in at least 50% of individuals). There are an additional 13 core OTUs not represented in the set of abundant OTUs. These 38 core and abundant OTUs include just six *de novo* OTUs not represented in the Greengenes database. Two of these are classified as *Wolbachia* (denovo7 and denovo8), three are classified as Lactobacillales (denovo9, denovo32, denovo63), and one is classified as Enterobacteriaceae (denovo1). The three Lactobacillales are 97% or more similar to a 16S sequence previously recovered from flower nectar (GenBank ID: KX656663). The closest hit in GenBank for denovo1 is the *Sodalis* endosymbiont of the weevil *Sitophilus oryzae* (CP006568) with 93% identity.

***Halictid bacterial communities distinct from other bees***

We compared the sequences recovered from halictids to two previous bee bacterial community datasets to determine the degree of overlap. Although our halictid samples and the honey bee samples from the previous dataset obtained from Qiita share 588 OTUs, of the 25 and 43 OTUs that make up at least 0.1% of the sequences from each dataset, respectively, only two overlap (GG833659: Pseudomonadales, GG4416113: Enterobacteriales). We also compared directly to the data from Martinson et al. (2011) which, though not collected using Illumina approaches, is still a valuable resource for assessing the similarity with other bee species. A total of 39 OTUs are shared between the current study's samples and those examined in Martinson et al. (2011) including seven that make up at least 0.1% of total sequences in each study (GG161287: Entomoplasmatales, GG6118: Rickettsiales, GG793101: Rhodospirillales, GG829017: Lactobacillales, GG833659: Pseudomonadales, GG835499: Rickettsiales, GG836919: Rickettsiales).

We also identified those OTUs present in at least 50% of all samples of *Lasioglossum leucozonium, L. laticeps, L. malachurum, L. pauxillum*, and *L. nigripes*. These species were chosen because they are all represented by at least 10 samples in our dataset. Between 26 (*L. malachurum*) and 69 (*L. laticeps*) core OTUs were identified in each species and between 4% (*L. malachurum* and 59% (*L. nigripes*) of these were not already represented in the set of halictid core OTUs. Taken together, there were 91 additional core OTUs from these species in addition to those identified as core or abundant OTUs in the overall halictid sampling. The resulting 129 OTUs were used for all comparisons of abundance between bee groups.

***Few differences in abundance***

Among those additional taxa that were identified as core members of species-level communities but were not core taxa among all halictids, 15 were significantly more abundant in solitary samples (FDR-corrected P < 0.01; Table S2). These include nine OTUs classified as Enterobacteriaceae*,* five OTUs classified as *Sodalis,* one classified as *Erwinia,* and one classified as *Wolbachia*, many of which were completely absent from social bees (Table S2)*.* No OTUs were significantly more abundant in social samples. Unfortunately, the small number of samples in which many of these taxa were present prevented the inclusion of geographic region in the model.

We implemented Mann-Whitney *U* tests between social forms of the polymorphic species *L. albipes*. A single OTU (GG830148: Lactobacillales) was significantly more abundant in social samples after FDR correction (P < 0.01). No taxa were significantly more abundant in solitary samples. Unfortunately, we are unable to control for geographical differences in *L. albipes* because differences in social behavior occur across populations in this species, and thus covary with geography.

We also used Mann-Whitney *U* tests of the copies of bacterial 16S rRNA gene per nanogram of DNA to test for differences in total quantity of bacteria in social and solitary samples. Quantities did not differ between social and solitary bees (P > 0.1) or between either social or solitary bees and parasitic bees. In general, the abundance of bacteria does not show any obvious pattern with bee behavior or evolutionary history (Figs. S2, S7). As expected, we recover a higher relative concentration of bacterial DNA in guts than whole abdomens (P = 0.016).

**Wolbachia *contributes to community differences***

The results when classifying communities from five bee species represented by at least 10 samples in our dataset had an error ratio of 2.71. However, removing the 127 *Wolbachia* and *Rickettsia* OTUs decreased the error ratio to 1.57. A classifier based entirely on the 127 *Wolbachia* and *Rickettsia* OTUs was quite successful with an error ratio of 2.42. Therefore, there is significant species-specificity of *Rickettsia* and, to a lesser extent, *Wolbachia.* While few recovered OTUs are unique to particular species, there are clear differences in frequency of individual taxa (Fig. S8). Results of all supervised learning analyses conducted are presented in Table S5.

A number of additional supervised learning analyses were conducted to further isolate the bacterial taxa most predictably associated with different groups of bees. For the comparison of social and solitary bees, limiting the supervised learning analysis to the family Enterobacteriaceae, rather than just the genus *Sodalis*, yields similar results (error ratio of 3.52).

The error ratio was 1.63 for classification by the 4 common geographic locations. Although this classifier fails to distinguish geographic regions, it is notable the top 3 OTU’s that are most important for this classifier are Lactobacillales. This provides some support for the conclusion that geography may be responsible for the single taxon significantly different in abundance between *L. albipes* behavioral types, though a classifier built exclusively on Lactobacillales taxa fails to distinguish regions (error ratio 1.49). The error ratio for the classifier of genera was 1.07, suggesting that little, if any, consistent differences exist between *Halictus* and *Lasioglossum*. Lastly, the supervised learning classifier was completely unable to distinguish between gut communities and abdomen communities (error ratio 1.33) strongly indicating that abdomens are equivalent to guts when characterizing bacterial communities from these bees.

We also explored the possibility of examining the absolute abundances of bacteria in these communities by multiplying the proportion of the number of copies of bacterial DNA found with qPCR per nanogram of DNA by the proportion of communities occupied by each OTU. This approach decreased the ability of machine learning to distinguish between species (error ratio of 1.31) and social behaviors (error ratio 2.17).

***Variation in alpha diversity***

We find that, when including all *Lasioglossum* samples, OTU counts are greater in solitary than social samples (Wilcoxon rank-sum test P = 0.01). The Chao1 index also supports this trend (P = 0.04). We, therefore, tested for differences in alpha diversity between individual pairs of social and solitary bee species with at least 10 specimens as including multiple species in a group is likely to impact alpha diversity measures. All tests between the solitary *L. leucozonium* and social taxa (*L. malachurum, L. laticeps, L. pauxillum,* and *L. nigripes*) are not significant (P > 0.05) except for *L. malachurum* (P = 7.9x10^-5^) for OTU counts and for Chao1 (P = 0.0005).

We also tested for differences in variance of alpha diversity in social and solitary samples using Brown-Forsythe tests. When including all *Lasioglossum* specimens, both OTU counts (F = 4.7, P = 0.04) and Chao1 (F = 4.7, P = 0.04) appear to have greater variance in solitary samples. However, when testing between individual species, these tests are not significant (P > 0.05) except between *L. leucozonium* and *L. malachurum* for both OTU counts (F = 22, P = 5.66x10^-5^) and Chao1 (F = 15.3, P = 0.0005). Evidence for greater variance in alpha diversity of solitary bees is, therefore, extremely weak.

**Sodalis *assembly and phylogeny***

None of the non-halictid bee data yielded consensus sequences with less than 95% missing data and all were 99.9% missing except for *C. calcarata* which had 2.3% complete consensus sequence. Because the distant relatedness of this bee species suggested that it might be particularly informative for understanding the *Sodalis* phylogeny, we included it in our inference despite the relatively small amount of data available.

The lineage inhabiting *C. calcarata* was distantly related to all others, closer to the *Sodalis* symbiont of tsetse flies than to any halictid-associated *Sodalis* (Fig. S4). The *L. leucozonium* lineage was also unique among the samples we examined and we named this *Sodalis* lineage “SLEU”. The rest of the samples fell into two large clades. The first (“SAL1”) includes 12 *L. albipes* samples as well as all of the other halictid samples*.* The last halictid-associated clade (“SAL2”) is composed entirely of *L. albipes* samples. Based on these results, we used metagenomic approaches to assemble genomes *de novo* for SLEU, SAL1, and SAL2.

Based on the phylogeny, we assembled four *Sodalis* genomes *de novo.* Firstly, SAL1 and SAL2 were assembled from individual *L. albipes* samples with the largest number of reads mapping to *S. pierantonius.* This was KMT2L-E12 for SAL1 with 19 million reads (~900x) and KMT2L-G10 for SAL2 with 3.3 million reads (~150x). The samples with the second-most numbers of reads were SDK-photo-03 for SAL1 and KMT3L-A1 for SAL2 and were used in multi-metagenome for differential coverage binning of genomic scaffolds. For the final SPAdes assemblies from the unmapped *L. albipes* data, the assembly at 240X coverage was most contiguous for SAL1 while for SAL2 the assembly at 25X was most contiguous. We also attempted to assemble genomes for each of the 10x Genomics datasets with large numbers of *Sodalis* reads but were only successful for *L. leucozonium* and *L. calceatum*. Similarly, we made efforts to assemble the *C. calcarata* inhabitant but were unsuccessful, never recovering a scaffold longer than 7kb. This is likely in part because the *C. calcarata* datasets that contained *Sodalis* sequence were not paired-end.

We then included SLEU, SAL1, and SAL2 in the ASTRAL species tree analysis which included 3,203 orthologous groups and yielded a consensus tree with a normalized score of 0.884. Posterior probabilities were 1.0 for every node in this consensus. We reconstructed another phylogeny by realigning the initial genomes with the four newly assembled bee-derived genomes and again mapping reads to the aligned *So. pierantonius* sequence, constraining the topology to the ASTRAL result. The *L. calceatum* derived *Sodalis* and SAL1 diverged by only 0.3% in this analysis and were, therefore, merged using Quickmerge and considered to be a single lineage for subsequent analyses (Fig. S4). All raw sequence reads used for the assembly of *Sodalis* genomes and for inferring the *Sodalis* phylogeny have been deposited in NCBI’s Short Read Archive under BioProject accession PRJNA402054.

**Sodalis *genome characteristics***

Separately assembling data from each identified clade of *Sodalis* yielded unique genome sequences. SAL1 was composed of 20 scaffolds totaling 4.1 Mb (though 527 kb are composed of gaps) and the largest scaffold making up 3.2 Mb of that assembly. RAST annotated 4,314 genes in this genome and it has an average GC-content of 56.7%. SAL2 has a similar total length of 4.2 Mb but these are placed in 241 scaffolds, the largest of which is 83 kb with an N50 size of 27 kb and a GC-content of 57.5%. Only 69 sites in this assembly are uncalled. There are 4,518 genes in this assembly. Lastly, the SLEU assembly is much smaller with a total length of 2.4 Mb on 86 scaffolds and the largest scaffold is 530 kb with an N50 of 372 kb, though 878 kb are gaps. RAST identified 2,404 genes in this genome and it has a %GC of 54.4. By comparison the free-living form of *Sodalis*, *So. praecaptivus*, has a GC-content of 57.5%, a length of 4.7 Mb and 4,411 genes annotated by RAST. Particularly for SLEU, the decreased length and GC-content in the halictid inhabitants both suggest that these genomes may be degenerating.

All three genomes were relatively complete. SAL2 has 98.8% of the expected 137 marker genes, SAL1 has 98.2%, and SLEU has 92.8%. Interestingly, coding density was also found to vary widely. Over the whole sequence, 74% of the SAL1 genome is composed of coding sequence, 84% of SAL2 is coding sequence, and only 58% of SLEU is coding sequence.

***No evidence for long-term vertical transmission***

We first identified the scaffold corresponding to the mitochondrial genome in the *L. albipes* genome [14] by querying the honey bee mitochondrial proteins against the *L. albipes* genome assembly. Of the 13 mitochondrial proteins, 10 had significant (e-value < 1) hits in the *L. albipes* assembly. Of these 10, all but one were best hits to scaffold2258. In the one exception, the difference between the e-values for the best hit and the hit to this scaffold was only 1.23x10^-13^. Of the 36 *L. albipes* with sufficient numbers of reads of *Sodalis* origin to be included in the phylogenetic analysis, we were able to identify mitochondrial haplotypes for 33 of them. Six unique haplotypes were identified with between two and 16 bees assigned to each. Haplotypes were never present in multiple geographic populations, though these populations did include multiple haplotypes (Fig. S4). The two largest haplotype groups representing all samples from Ventoux (16 bees) and the majority of samples from Vosges (seven bees), included samples within both *Sodalis* lineages. In the Ventoux group, most bees were occupied by SAL2 but two bees were dominated by SAL1 and in the Vosges haplotype, three bees were dominated by SAL1 and four were dominated by SAL2. Though this does not exclude the possibility of maternal transmission of *Sodalis*, it does not provide any support for it.

**Sodalis *is present throughout the body***

We conducted diagnostic PCR’s for the presence of *Sodalis* in three *L. albipes* abdomens, thoraces, heads, legs, and antennae. These yielded a product for all body parts for all three samples, except for heads. The amplification of bee COI sequence was also unsuccessful for heads indicating that this DNA extract was likely of too low quality for PCR. While their abundances likely vary in different body parts, these results suggest that *Sodalis* are found throughout the body and are likely present within the hemolymph of bees, as seen for *So. glossinidius,* the *Sodalis* symbiont of tsetse flies [40,41] and a number of other vertically transmitted, non-gut symbionts.

We addressed the possibility that *Sodalis* was an environmentally-derived contaminant present on the exoskeletons of the samples studied by comparing abundance of taxa within whole-abdomens to the 30 gut dissections. We tested each of the most abundant and core OTUs for differences in abundance between guts and abdomens using Mann-Whitney *U* tests to determine whether they are likely associated with the gut. Three taxa were significantly more abundant in abdomens than guts after FDR correction (GG829017: Lactobacillales, GG830148: Lactobacillales, and GG273974: *Wolbachia*; P < 0.01). *Sodalis* taxon GG4316320 was more abundant in guts alone (P = 0.049; mean of 4,078 vs 1,551 reads after rarefying to 20,000). The other three *Sodalis* taxa were also not significantly different in abundance (P > 0.1). This pattern suggests that much of the *Sodalis* population present in these samples is restricted to the gut and is not restricted to other organs or to the cuticle.

**Supplementary figures**

**Figure S1.**

Sites from which samples were collected. Barplots show the average bacterial community composition for social and solitary samples collected from each site. The three most common bacterial taxa are colored and all other taxa are shown in grayscale. Histograms show the proportions of shotgun sequencing reads from *L. albipes* samples from six locations (Calais, Dordogne, Rimont, Vosges, Brassus, and Ventoux) that mapped to *Sodalis*.

**Figure S2.**

A. Distributions of community dispersion in all social and solitary samples as well as those species represented by at least 10 individuals. The polymorphic *L. albipes* is split into social and solitary categories (n=19 solitary, n=6 social). Dispersions are not significantly different between social and solitary bees based on Wilcoxon rank-sum tests. The same test was used to compare all strictly social species to the strictly solitary species *L. leucozonium* and, again, none were significant. Data for *L. albipes* are included for reference; they were not included in statistical comparisons. B. Boxplots showing the number of copies of the bacterial 16S rRNA gene per nanogram of DNA.

**Figure S3.**

Heatmap of the presence or absence of *Sodalis* classified OTUs that appear in at least 5 samples in all *Lasioglossum* used in the comparison of social and solitary samples.

**Figure S4.**

Phylogeny of recovered *Sodalis* sequences. Bolded taxa represent genomes assembled *de novo* in this study. *C. calcarata,* *L. vierecki*, *L. marginatum*, *L. malachurum*, and *H. ligatus* all represent *Sodalis* sequences recovered from shotgun sequencing libraries derived from those bee taxa. *So. glossinidius* and *So. pierantonius* are previously assembled symbionts of tsetse flies and weevils, respectively. *So. praecaptivus* is the previously assembled free-living form of *Sodalis*. All other tips are derived from *L. albipes* shotgun sequencing libraries and are labeled with their location of origin and mitochondrial haplotype in parentheses. Each number represents a unique haplotype. We were not able to recover haplotypes for all samples.

**Figure S5.**

Proportion of genes belonging to each functional subsystem in each of the *Sodalis* lineages discovered in halictids relative to the number of genes identified in the closely-related free-living *So. praecaptivus*. Subsystem membership was determined using RAST and numbers of genes classified to each function in *So. praecaptivus* are given in parentheses.

**Figure S6.**

Rarefaction curves for all halictids samples examined in this study.

**Figure S7.**

Heatmap of relative proportions of the 24 most common bacterial taxa across all samples. When a particular OTU is present in a sample at less than 0.01% frequency, it is colored dark gray. Samples are grouped by genus and social behavior. *Lasioglossum* and *Halictus* samples without a specified behavior are from samples with unknown sociality. Bacterial phylogeny and taxonomy are derived from Greengenes.

**Figure S8.**

Boxplots show the quantity of bacteria present in each sample as determined by qPCR of the bacterial 16S rRNA gene. Polymorphic taxa are colored by the behavior of the samples included.

**Figure S9.**

Heatmap of the presence or absence of *Wolbachia* and *Rickettsia* classified OTUs that appear in at least five individuals in the five *Lasioglossum* species used in the between-species comparisons. Species boundaries are somewhat apparent based on the presence of these bacterial taxa.

**Supplementary tables**

**Table S1.**

Amplicon sequencing sample information.

**Table S2.**

Statistical comparisons of core taxa in social and solitary samples.

**Table S3.**

Full OTU table.

**Table S4.**

Shotgun sequencing sample information.

**Table S5.**

Summary of all supervised learning analyses conducted.

**References**

1. Rubin BER, Sanders JG, Hampton-Marcell J, Owens SM, Gilbert JA, Moreau CS. 2014 DNA extraction protocols cause differences in 16S rRNA amplicon sequencing efficiency but not in community profile composition or structure. *MicrobiologyOpen* **3**, 910–921. (doi:10.1002/mbo3.216)

2. Sanders JG, Łukasik P, Frederickson ME, Russell JA, Koga R, Knight R, Pierce NE. 2017 Dramatic differences in gut bacterial densities correlate with diet and habitat in rainforest ants. *Integr. Comp. Biol.* (doi:10.1093/icb/icx088)

3. Caporaso JG *et al.* 2010 QIIME allows analysis of high-throughput community sequencing data. *Nat. Methods* **7**, 335–336. (doi:10.1038/nmeth.f.303)

4. Edgar RC. 2013 UPARSE: highly accurate OTU sequences from microbial amplicon reads. *Nat. Methods* **10**, 996–998. (doi:10.1038/nmeth.2604)

5. Martinson VG, Danforth BN, Minckley RL, Rueppell O, Tingek S, Moran NA. 2011 A simple and distinctive microbiota associated with honey bees and bumble bees. *Mol. Ecol.* **20**, 619–628. (doi:10.1111/j.1365-294X.2010.04959.x)

6. Cole JR *et al.* 2014 Ribosomal Database Project: data and tools for high throughput rRNA analysis. *Nucleic Acids Res.* **42**, D633–D642. (doi:10.1093/nar/gkt1244)

7. Caporaso JG, Bittinger K, Bushman FD, DeSantis TZ, Andersen GL, Knight R. 2010 PyNAST: a flexible tool for aligning sequences to a template alignment. *Bioinformatics* **26**, 266–267. (doi:10.1093/bioinformatics/btp636)

8. DeSantis TZ *et al.* 2006 Greengenes, a chimera-checked 16S rRNA gene database and workbench compatible with ARB. *Appl. Environ. Microbiol.* **72**, 5069–5072. (doi:10.1128/AEM.03006-05)

9. Gibbs J, Brady SG, Kanda K, Danforth BN. 2012 Phylogeny of halictine bees supports a shared origin of eusociality for *Halictus* and *Lasioglossum* (Apoidea: Anthophila: Halictidae). *Mol. Phylogenet. Evol.* **65**, 926–939. (doi:10.1016/j.ympev.2012.08.013)

10. Plateaux-Quénu C. 2008 Subsociality in halictine bees. *Insectes Sociaux* **55**, 335–346. (doi:10.1007/s00040-008-1028-z)

11. Navas-Molina JA *et al.* 2013 Advancing our understanding of the human microbiome using QIIME. In *Methods in Enzymology*, pp. 371–444. Elsevier. (doi:10.1016/B978-0-12-407863-5.00019-8)

12. Van Treuren W *et al.* 2015 Variation in the microbiota of *Ixodes* ticks with regard to geography, species, and sex. *Appl. Environ. Microbiol.* **81**, 6200–6209. (doi:10.1128/AEM.01562-15)

13. Schloss PD *et al.* 2009 Introducing mothur: Open-source, platform-independent, community-supported software for describing and comparing microbial communities. *Appl. Environ. Microbiol.* **75**, 7537–7541. (doi:10.1128/AEM.01541-09)

14. Kocher SD *et al.* 2013 The draft genome of a socially polymorphic halictid bee, *Lasioglossum albipes*. *Genome Biol* **14**, R142. (doi:10.1186/gb-2013-14-12-r142)

15. Li H, Durbin R. 2009 Fast and accurate short read alignment with Burrows-Wheeler transform. *Bioinformatics* **25**, 1754–1760. (doi:10.1093/bioinformatics/btp324)

16. Garrison E, Marth G. 2012 Haplotype-based variant detection from short-read sequencing. *ArXiv Prepr. ArXiv12073907*

17. Stamatakis A. 2006 RAxML-VI-HPC: maximum likelihood-based phylogenetic analyses with thousands of taxa and mixed models. *Bioinformatics* **22**, 2688–2690. (doi:10.1093/bioinformatics/btl446)

18. Luo R *et al.* 2012 SOAPdenovo2: an empirically improved memory-efficient short-read de novo assembler. *Gigascience* **1**, 18. (doi:10.1186/2047-217X-1-18)

19. Boisvert S, Raymond F, Godzaridis É, Laviolette F, Corbeil J, others. 2012 Ray Meta: scalable de novo metagenome assembly and profiling. *Genome Biol* **13**, R122. (doi:10.1186/gb-2012-13-12-r122)

20. Peng Y, Leung HCM, Yiu SM, Chin FYL. 2012 IDBA-UD: a de novo assembler for single-cell and metagenomic sequencing data with highly uneven depth. *Bioinformatics* **28**, 1420–1428. (doi:10.1093/bioinformatics/bts174)

21. Scholz M, Lo C-C, Chain PSG. 2014 Improved assemblies using a source-agnostic pipeline for MetaGenomic Assembly by Merging (MeGAMerge) of contigs. *Sci. Rep.* **4**, 6480. (doi:10.1038/srep06480)

22. Albertsen M, Hugenholtz P, Skarshewski A, Nielsen KL, Tyson GW, Nielsen PH. 2013 Genome sequences of rare, uncultured bacteria obtained by differential coverage binning of multiple metagenomes. *Nat. Biotechnol.* **31**, 533–538. (doi:10.1038/nbt.2579)

23. Bankevich A *et al.* 2012 SPAdes: A new genome assembly algorithm and its applications to single-cell sequencing. *J. Comput. Biol.* **19**, 455–477. (doi:10.1089/cmb.2012.0021)

24. Nadalin F, Vezzi F, Policriti A. 2012 GapFiller: a *de novo* assembly approach to fill the gap within paired reads. *BMC Bioinformatics* **13**, S8. (doi:10.1186/1471-2105-13-S14-S8)

25. Buchfink B, Xie C, Huson DH. 2014 Fast and sensitive protein alignment using DIAMOND. *Nat. Methods* **12**, 59–60. (doi:10.1038/nmeth.3176)

26. Chakraborty M, Baldwin-Brown JG, Long AD, Emerson JJ. 2016 Contiguous and accurate *de novo* assembly of metazoan genomes with modest long read coverage. *Nucleic Acids Res.* **44**, e147. (doi:10.1093/nar/gkw654)

27. Adey A *et al.* 2014 *In vitro*, long-range sequence information for *de novo* genome assembly via transposase contiguity. *Genome Res.* **24**, 2041–2049. (doi:10.1101/gr.178319.114)

28. Parks DH, Imelfort M, Skennerton CT, Hugenholtz P, Tyson GW. 2015 CheckM: assessing the quality of microbial genomes recovered from isolates, single cells, and metagenomes. *Genome Res.* **25**, 1043–1055. (doi:10.1101/gr.186072.114)

29. Aziz RK *et al.* 2008 The RAST Server: Rapid Annotations using Subsystems Technology. *BMC Genomics* **9**, 75. (doi:10.1186/1471-2164-9-75)

30. Lechner M, Findeis S, Steiner L, Marz M, Stadler PF, Prohaska SJ. 2011 Proteinortho: detection of (co-) orthologs in large-scale analysis. *BMC Bioinformatics* **12**, 124. (doi:10.1186/1471-2105-12-124)

31. Loytynoja A, Goldman N. 2008 A model of evolution and structure for multiple sequence alignment. *Philos. Trans. R. Soc. B Biol. Sci.* **363**, 3913–3919. (doi:10.1098/rstb.2008.0170)

32. Mirarab S, Warnow T. 2015 ASTRAL-II: coalescent-based species tree estimation with many hundreds of taxa and thousands of genes. *Bioinformatics* **31**, i44–i52. (doi:10.1093/bioinformatics/btv234)

33. Mirarab S, Reaz R, Bayzid MS, Zimmermann T, Swenson MS, Warnow T. 2014 ASTRAL: genome-scale coalescent-based species tree estimation. *Bioinformatics* **30**, i541–i548. (doi:10.1093/bioinformatics/btu462)

34. Sayyari E, Mirarab S. 2016 Fast coalescent-based computation of local branch support from quartet frequencies. *Mol. Biol. Evol.* **33**, 1654–1668. (doi:10.1093/molbev/msw079)

35. Yang Z. 1997 PAML: a program package for phylogenetic analysis by maximum likelihood. *Comput. Appl. Biosci. CABIOS* **13**, 555–556.

36. Yang Z. 2007 PAML 4: phylogenetic analysis by maximum likelihood. *Mol. Biol. Evol.* **24**, 1586–1591. (doi:10.1093/molbev/msm088)

37. DePristo MA *et al.* 2011 A framework for variation discovery and genotyping using next-generation DNA sequencing data. *Nat. Genet.* **43**, 491–498. (doi:10.1038/ng.806)

38. McKenna A *et al.* 2010 The Genome Analysis Toolkit: A MapReduce framework for analyzing next-generation DNA sequencing data. *Genome Res.* **20**, 1297–1303. (doi:10.1101/gr.107524.110)

39. Van der Auwera GA *et al.* 2013 From FastQ Data to High-Confidence Variant Calls: The Genome Analysis Toolkit Best Practices Pipeline: The Genome Analysis Toolkit Best Practices Pipeline. In *Current Protocols in Bioinformatics* (eds A Bateman, WR Pearson, LD Stein, GD Stormo, JR Yates), p. 11.10.1-11.10.33. Hoboken, NJ, USA: John Wiley & Sons, Inc. (doi:10.1002/0471250953.bi1110s43)

40. Balmand S, Lohs C, Aksoy S, Heddi A. 2013 Tissue distribution and transmission routes for the tsetse fly endosymbionts. *J. Invertebr. Pathol.* **112**, S116–S122. (doi:10.1016/j.jip.2012.04.002)

41. Cheng Q, Aksoy S. 1999 Tissue tropism, transmission and expression of foreign genes *in vivo* in midgut symbionts of tsetse flies. *Insect Mol. Biol.* **8**, 125–132.
