# Supplementary figures and images for: Social behaviour in bees influences the abundance of Sodalis (Enterobacteriaceae) symbionts
Source: R Soc Open Sci. 2018 Jul 11;5(7):180369. doi: 10.1098/rsos.180369 (PMC6083661; doi:10.1098/rsos.180369)

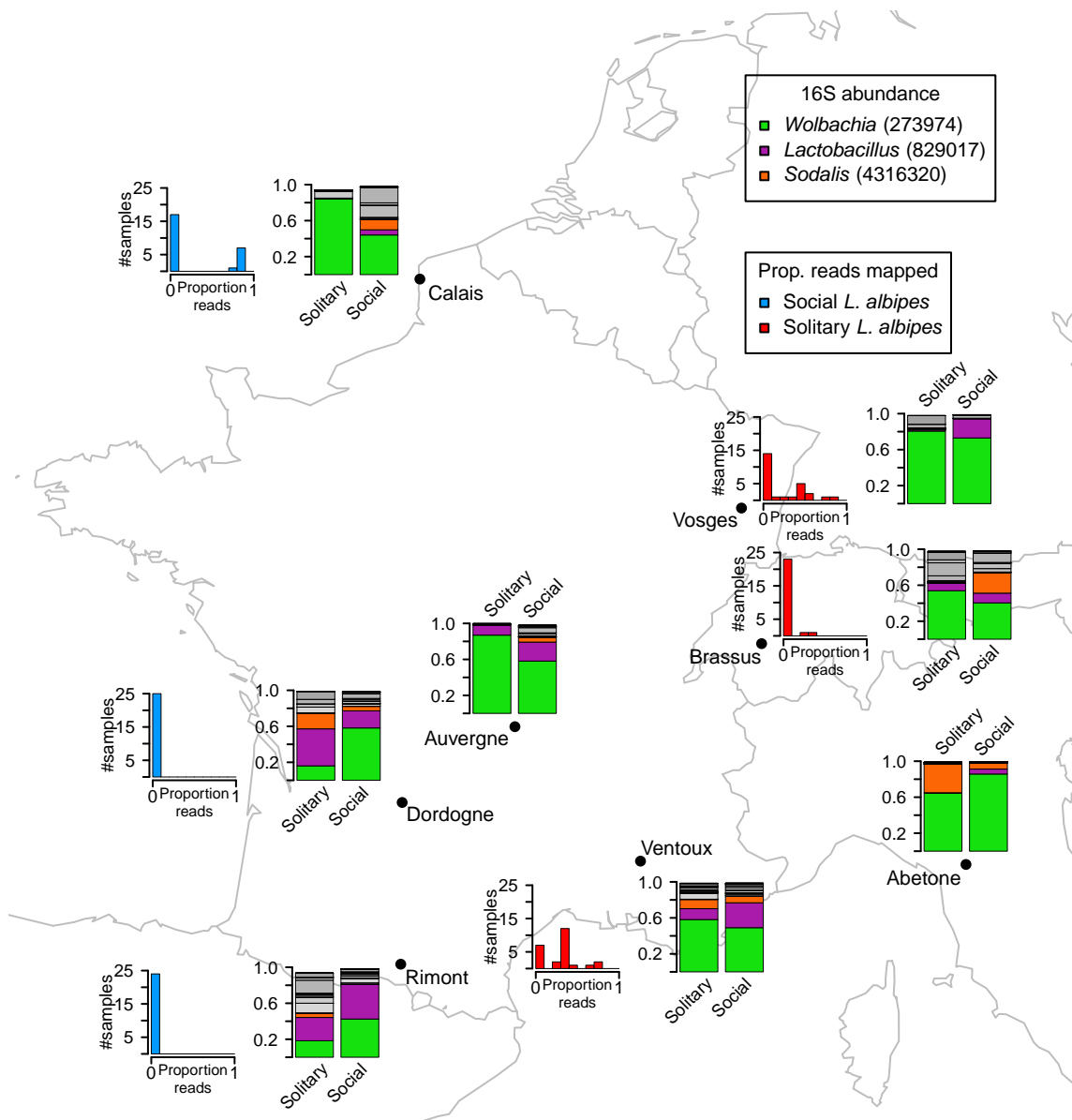

Supplement: Figure S1 [file rsos180369supp2.pdf]

**A**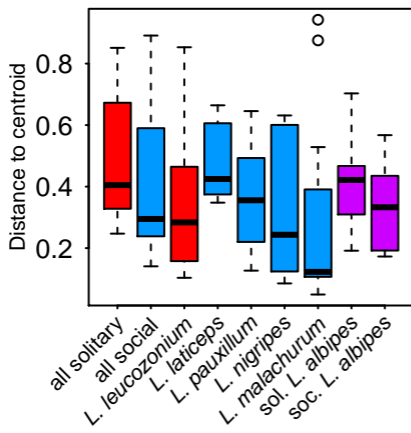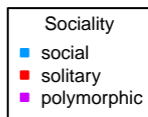**B**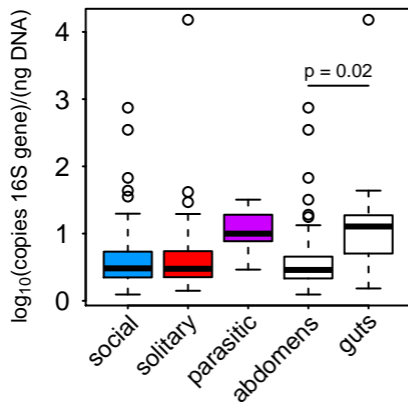

Supplement: Figure S2 [file rsos180369supp3.pdf]

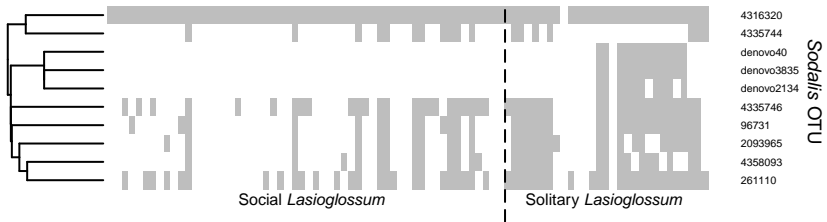

Supplement: Figure S3 [file rsos180369supp4.pdf]

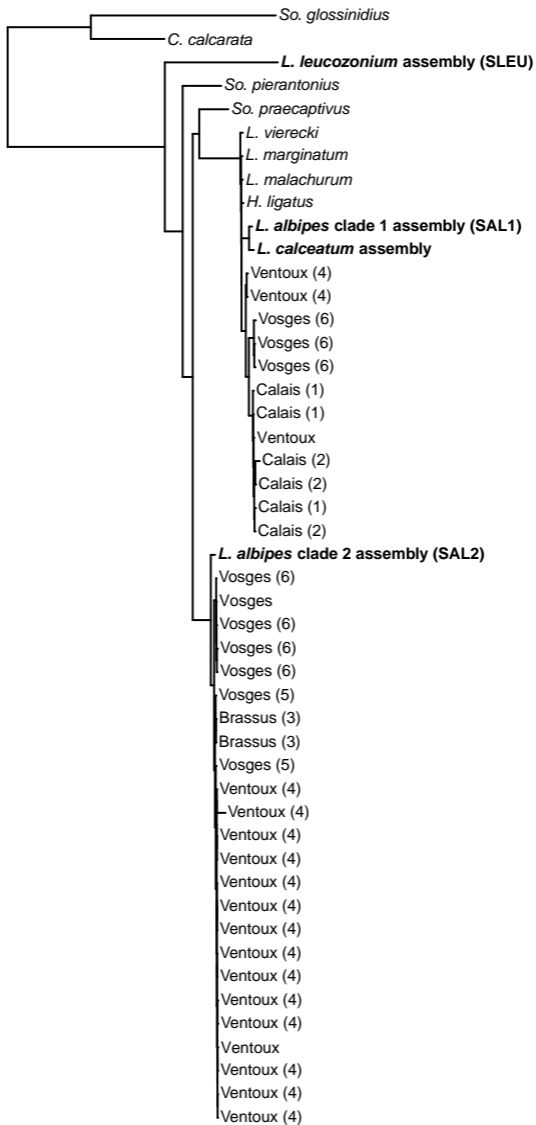

0.02 substitutions/site —

Supplement: Figure S4 [file rsos180369supp5.pdf]

Proportion genes present

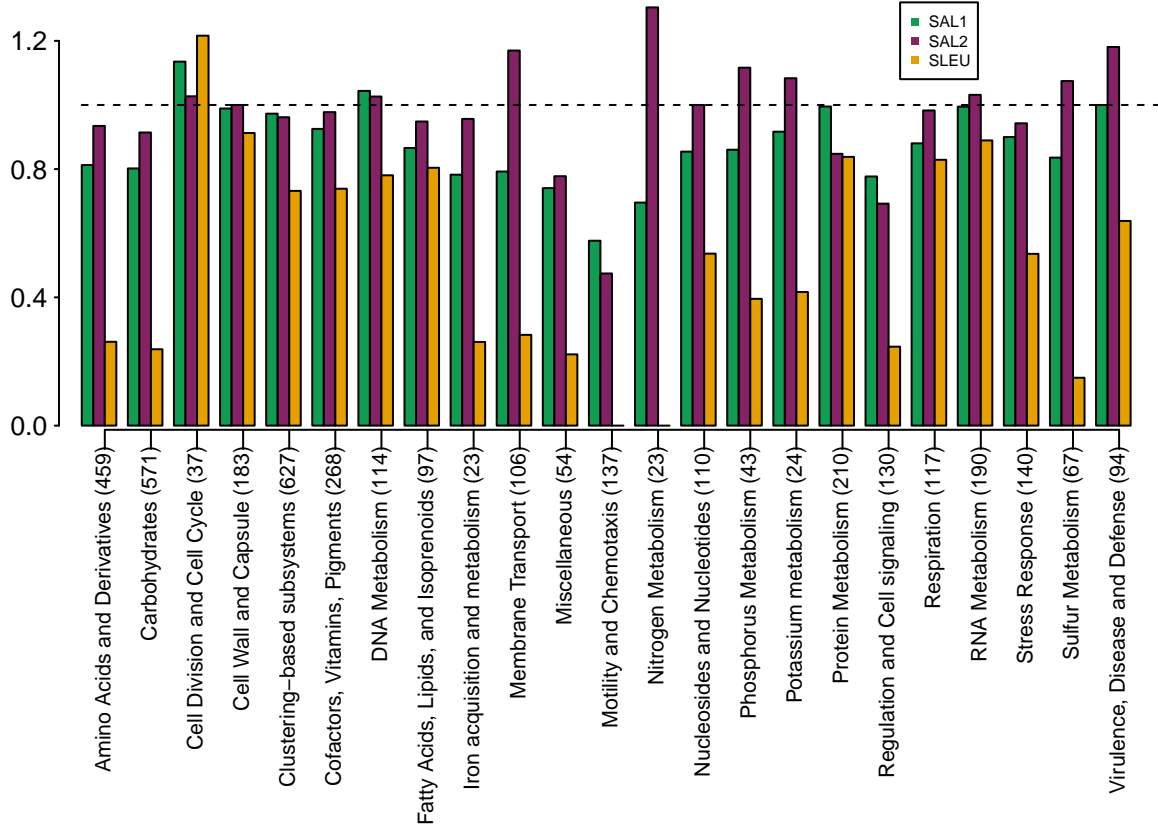

Supplement: Figure S5 [file rsos180369supp6.pdf]

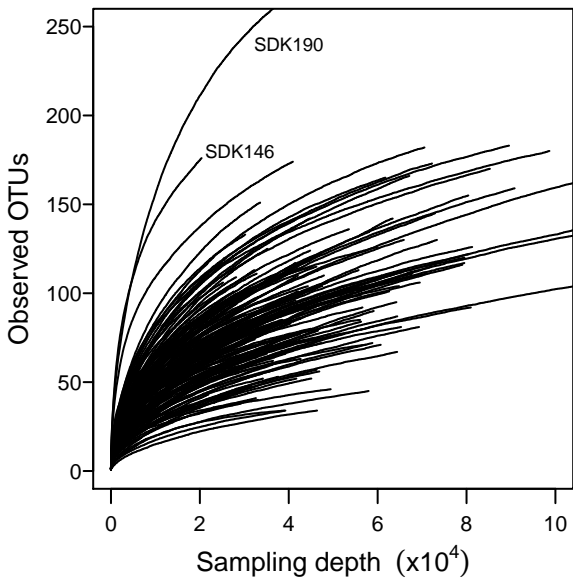

Supplement: Figure S6 [file rsos180369supp7.pdf]

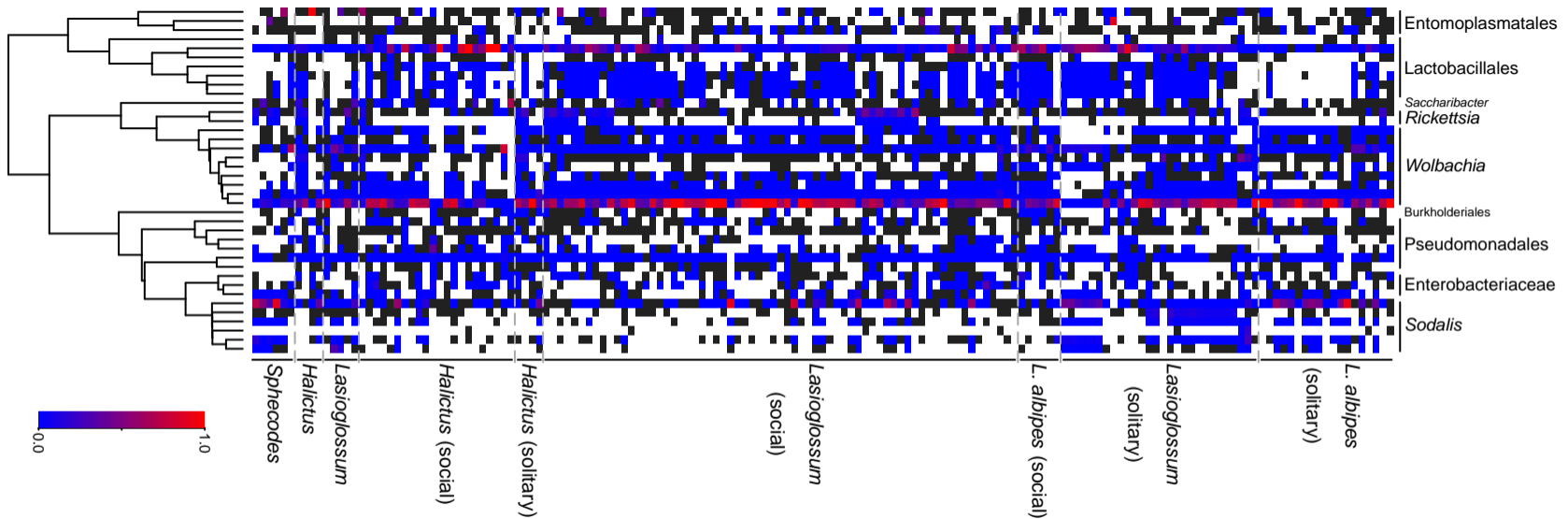

Supplement: Figure S7 [file rsos180369supp8.pdf]

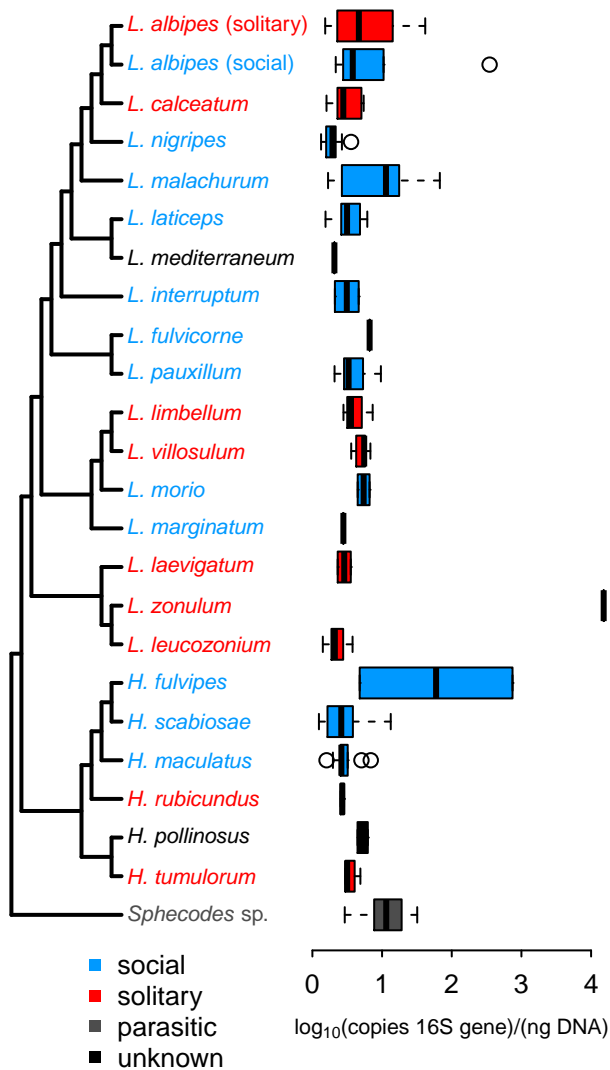

Supplement: Figure S8 [file rsos180369supp9.pdf]

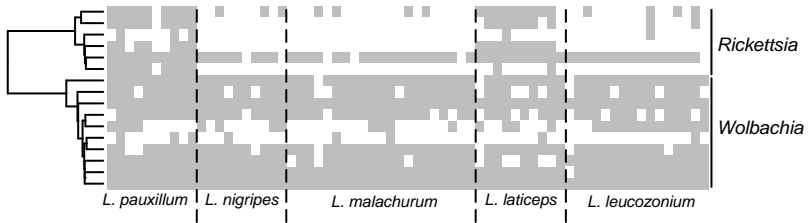

Supplement: Figure S9 [file rsos180369supp10.pdf]
